# Autoantibodies in Morphea: An Update

**DOI:** 10.3389/fimmu.2019.01487

**Published:** 2019-07-09

**Authors:** Sangita Khatri, Kathryn S. Torok, Emily Mirizio, Christopher Liu, Kira Astakhova

**Affiliations:** ^1^Department of Chemistry, Technical University of Denmark, Kongens Lyngby, Denmark; ^2^Division of Rheumatology, Department of Pediatrics, Children's Hospital of Pittsburgh, University of Pittsburgh, Pittsburgh, PA, United States

**Keywords:** skin autoimmunity, morphea, autoantibody, diagnostics, personalized management

## Abstract

Skin autoimmune conditions belong to a larger group of connective tissue diseases and primarily affect the skin, but might also involve underlying tissues, such as fat tissue, muscle, and bone. Autoimmune antibodies (autoantibodies) play a role in autoimmune skin diseases, such as localized scleroderma also termed morphea, and systemic scleroderma, also called systemic sclerosis (SSc). The detailed studies on the biological role of autoantibodies in autoimmune skin diseases are limited. This results in a few available tools for effective diagnosis and management of autoimmune skin diseases. This review aims to provide an update on the detection and most recent research on autoantibodies in morphea. Several recent studies have indicated the association of autoantibody profiles with disease subtypes, damage extent, and relapse potential, opening up exciting new possibilities for personalized disease management. We discuss the role of existing autoantibody tests in morphea management and the most recent studies on morphea pathogenesis. We also provide an update on novel autoantibody biomarkers for the diagnosis and study of morphea.

## Introduction

Being a part of an abnormal immune response, autoimmune antibodies (autoantibodies) are valuable biomarkers in autoimmunity. The causality of autoantibodies in autoimmune diseases is still controversial and requires more fundamental research (1). Nevertheless, clinical assays detecting autoantibodies are commonly used to diagnose and categorize autoimmune diseases (2). Recent studies have revealed distinct autoantibody profiles among patients with autoimmune diseases, opening up new avenues for better diagnostics and personalized disease management (3).

The prototypical rheumatologic autoimmune disease of the skin, systemic sclerosis (SSc), is often defined and subcategorized by an auto-antibody profile, in addition to the extent of skin involvement (4). The presence of scleroderma-associated autoantibodies, such as autoantibodies to topoisomerase and centromeres, has enabled clinicians to better predict organ system involvement in these patients. Nevertheless, the true pathogenicity of these autoantibodies in SSc disease propagation remains to be elucidated.

**Figure 1:**
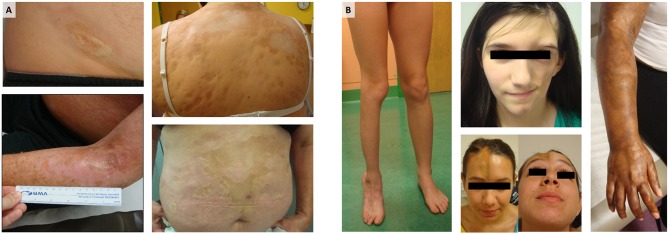
Morphea: **(A)** Generalized morphea and plaque morphea are the most common subtypes of morphea in adult patients, while **(B)** linear scleroderma of the trunk, limbs, and head are the most common in pediatric morphea. For each photograph, written informed consent was obtained from the participant for the publication of this image.

A “sister” autoimmune disease to SSc in regards to its similar effect on the skin is localized scleroderma, also termed morphea. Although morphea and SSc share the same skin histopathologic changes, the distribution and pattern of skin involvement, and the associated extracutaneous and internal organ manifestations, are quite different (5). Morphea is typically distributed in patches or bands of skin inflammation and thickness either on the head, extremity, and trunk in an ipsilateral fashion (Figure 1). Based on the distribution patterns and depth of lesions, morphea has been divided into several clinical subtypes: circumscribed superficial morphea (plaque morphea), superficial deep morphea (deep morphea), generalized plaque morphea (multiple plaque lesions), linear scleroderma of the trunk/extremities, linear scleroderma of the head (also termed Parry–Romberg Syndrome and En coup de sabre), and pansclerotic morphea or mixed morphea (a combination of two or more of these subtypes) (6). Although morphea does not tend to have internal organ manifestations, such as interstitial lung disease or cardiac arrhythmia, the underlying and associated tissue is often affected in morphea patients, causing morbidity. The frequency of extracutaneous involvement in morphea has ranged from 20 up to 70% in the literature, depending on whether the data has been collected retrospectively or prospectively, with prospective assessment data capturing more manifestations (7–13). The most common extracutaneous manifestations (ECM) across cohorts are musculoskeletal, including arthralgia, arthritis, joint contractures, myositis, fasciitis, with associated disease-induced gait disturbance, decreased function, muscle, bone, and limb atrophy (Figure 1), and followed by neurologic, ophthalmologic, and dental issues occurring in ~50% of morphea patients with involvement of the head (14–16). These manifestations include seizures, headaches, hemiparesis, cranial nerve palsy, optic neuritis, uveitis, scleritis, dry eye, atrophic dental roots, dental crowding, and malocclusion. Morphea subtypes and ECM associations are different in adult vs. childhood-onset. Circumscribed superficial (plaque morphea) and generalized morphea are common in adult-onset morphea (Figure 1A), while linear scleroderma, both linear trunk/extremity and linear head subtypes, is more common in childhood-onset morphea (Figure 1B) (10, 11). To achieve personalized management schemes, these factors shall be taken into account.

In general, morphea is monitored clinically through the visualization and scoring of skin changes over time (Figure 1). Several clinical assessment methods have been developed to monitor morphea, such as depigmentation, induration, erythema, and the telangiectasia (DIET) score, the modified Rodnan skin score (mRSS), and the Localized Scleroderma Assessment Tool (LoSCAT) (12, 17). All of these methods assess activity and damage together, based on selected clinical parameters. The lack of a full validation of treatment response criteria of these scores limits the ability of clinicians to judge the effectiveness of these treatments. A combination of a cutaneous outcome measure in conjunction with serological biomarkers, such as autoantibodies, might be a plausible means to better classify and stratify morphea patients.

With regard to serological testing, the role of autoantibodies in morphea and their clinical application is not as clear as SSc. So far, it is recommended to use the Localized Scleroderma Assessment Tool (LoSCAT), with autoantibody tests when concurrent rheumatic and other autoimmune diseases are suspected. Suggested tests to verify this include antinuclear antibodies (ANA), antibodies to single stranded DNA (a-ssDNA), anti-histone antibodies (AHA), and anti-topoisomerase antibody type of anti-nuclear autoantibodies (anti-Scl-70).

A particular limitation of the literature in regards to autoantibody testing in morphea is that the majority of studies reporting autoantibody positivity are in context of a larger descriptive morphea cohort summary; therefore only a subset of patients have available autoantibody testing, typically guided by clinical practice instead of prospective research testing. To address this limitation, we only included cohort studies in which at least 50 morphea subjects were tested for the autoantibody of interest, in addition to prospective studies designed to test autoantibodies in morphea, to obtain more accurate frequencies and clinical correlations.

The review is organized into three parts. First, we describe the status with existing autoantibody tests (ANA, AHA, and a-ssDNA) in the context of morphea diagnosis and treatment. Here, the literature has been selected based on the number of patients with a particular autoantibody tested within each cohort (≥50 patients), and suggested associations with clinical manifestations of the disease. In the second part, we present the most recent studies on the role of autoantibodies in the pathogenesis of morphea. Here, we selected studies that included relevant controls and suggested pertinent new knowledge on morphea pathogenesis. Third, we describe emerging biomarkers in morphea research and clinical diagnostics; in this part, we select the literature based on the statistical power of the observed effects, and the appeal to potential personalized management of morphea patients.

Besides a low number of enrolled patients, a large deviation in the applied methodologies is another main problem in the field of morphea diagnostics. Therefore, we critically assessed the methodology for autoantibody testing, applied controls, and standardization methods. We also discuss the problem of the limited number of relevant animal models for morphea research, and the connection between recent studies on the potential to reveal new valuable insights into the pathogenesis of morphea, and to improve the disease management in clinics.

## Laboratory Diagnostics of Autoantibodies in Morphea

### ANA Positivity in Morphea

**Figure 2:**
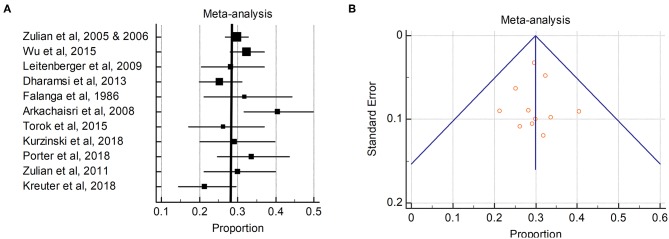
Pooled ANA positivity in patients with morphea: **(A)** The ANA positivity (percentages) in the individual studies are represented by squares, through which the horizontal lines represent the 95% CIs. The thick vertical line represents the pooled ANA positivity from these studies; **(B)** Funnel plot of the proportion vs. the standard error of the proportion for ANA positivity. The circles represent the trials included in the meta-analysis. The line in the center indicates the summary proportion. The other lines represent the 95% CIs. Asymmetry about the pooled proportion line is consistent with the presence of minimal publication bias.

Elevated antinuclear antibody (ANA) levels are often encountered in morphea, with larger cohort reports detecting 23–68% ANA-positive patients (see Figure 2; Supplementary Table 1 and cited references). The majority of studies (>80%) describe ANA testing by Human epithelial type 2 immunofluorescence assay (Hep2 IF), with a cut-off of >1:80 in most papers, run in standardized laboratory associated with the ordering institution. Notably, both pediatric-onset and adult-onset morphea have ANA positivity in this range (Supplementary Table 1 and cited references). One cohort study (Morphea in Adults and Children; MAC) reported a trend toward higher ANA positivity in adults (53% vs. 30% pediatrics) (10). However, in a later study with more subjects enrolled in the MAC cohort, the frequency of ANA positivity was higher in pediatric onset (27% in adults vs. 42% in pediatrics patients). The largest cohort to date with ANA testing is an international pediatric morphea cohort described by Zulian et al. in which 750 morphea patients were described, 671 in whom had ANA testing performed (13, 18). Of these, 284 patients (42.3%) were ANA positive. ANA positivity was tested among subtypes of morphea (linear, plaque, generalized, and deep morphea) and there was no significant difference (18), with subtype ANA positivity ranging from 31 to 47%. ANA positivity has been debated to be associated with the subtype of morphea, with the initial findings of the MAC cohort supporting an association with generalized morphea and mixed morphea (generalized + linear) (10). Their later study using matched healthy case-controls compared to the morphea cohort did not find an association of ANA positivity with morphea subtype (19).

A metanalyses of ANA positivity was performed to more formally capture the average ANA positivity in morphea patients across various cohort publications. Our search strategy aimed to identify studies that described antibody testing or cohort reports in morphea (localized scleroderma). All studies that reported antibody testing were reviewed (both retrospective and prospective). Independent searches of the PubMed, Google Scholar, and Webscope databases for relevant studies was performed using the following search terms: morphea, localized scleroderma, antibody, ANA, AHA, pediatric, rheumatoid factor (RF), juvenile, morphea cohort etc. The search was limited to studies that included 50 or more patients tested for antibodies of interest. Citations were screened for duplicate studies and duplicate patient samples without blinding. Statistics software (MedCalc Software, Ostend, Belgium) was used for the statistical analysis. Outcomes were measured by calculating proportions and 95% confidence intervals (CIs) for each study, then pooled the data to derive a pooled proportion and 95% CI.

For the purpose of proportion meta-analysis, the proportions were first turned into a quantity (the Freeman-Tukey variant of the arcsine square root transformed proportion) suitable for the usual fixed and random effects summaries (20, 21). The pooled proportion was calculated as the back transform of the weighted mean of the transformed proportions, using DerSimonian weights for the random effects model (22) in the presence of significant heterogeneity. The impact of heterogeneity on the pooled estimates of the individual outcomes of the meta-analysis was assessed with the Cochran Q statistic and *I*^2^ test, which measure the inconsistency between the study results, which was interpreted as the approximate proportion of total variation in the study estimates that was due to heterogeneity rather than sampling error (23). *I*^2^ values of 25, 50, and 75% are considered low, moderate, and high estimates, respectively (24). As the Cochran Q test has a low sensitivity for detecting heterogeneity, a *P-*value of < 0.1 was considered significant for the presence of statistical heterogeneity (25). The presence of publication bias was checked with the Begg funnel plot (26), which plots the proportion (in the X axis) against the standard error of the proportion (in the Y axis). In the absence of publication bias, the proportion estimates from smaller studies are expected to be scattered above and below the summary estimate, producing a triangular or funnel shape.

The ANA positivity in primarily pediatric onset morphea patients ranged from 5.9 to 68%. The pooled ANA positivity was 29.9% (95% CI 27.3–32.5%) by the random effects model (Figure 2). There was significant but low heterogeneity for ANA positivity (*I*^2^ 38.3%, Cochran *Q* statistic 16.2, *P* = 0.008). Subanalyses were performed in attempt to identify patient characteristics or variables which may influence the heterogeneity, such as sex and subtype. Female sex had non-significant (*p* = 0.21) effect on the ANA+ heterogeneity. Age of patient had a significant effect on ANA+ heterogeneity (*p* = 0.0001), with the pediatric onset cohorts having more consistent ANA+ frequency compared to adult cohorts. When dichotomizing morphea subtype into linear (linear extremity/trunk and head) vs. nonlinear (generalized, plaque, deep morphea), there was also a significant impact on ANA+ which was more moderate with *I*^2^ 68%.

Although not directly related to the morphea subtype, ANA-positivity does seem to associate with disease severity in regards to the depth of the disease, association with ECMs, as well as with the probability of the disease flare after remission. A study of a large pediatric international cohort (*n* = 750) reported a significant association of extracutaneous manifestations with ANA positivity (13). In this cohort, there were 168 patients (22%) with ECMs, the most common being articular (47%), followed by neurologic, vascular, ocular, and autoimmune (13). When comparing skin involvement with extracutaneous involvement subjects, the authors also noted that the erythrocyte sedimentation rate, C-reactive protein, creatinine kinase, serum IgG, and rheumatoid factor were significantly elevated in those with ECMs. Rheumatoid factor has been measured by ELISA; other clinical tests were done following standard clinical procedures utilizing pre-determined normal range for the age of the patient per test. Noteworthy, each of these laboratory tests was available for at least 350 patients, which is a limitation of several other studies. The authors argued that the positive ANA in morphea patients might be associated with either deeper tissue involvement or with a more immunogenic or true “systemic” autoimmune disease. The latter is typically associated with a myriad of positive immune activation markers (ANA, ESR, CRP, IgG, and other autoantibodies discussed next).

Data from the second largest morphea cohort published, the Childhood Arthritis and Rheumatology Research Alliance (CARRA) North American registry cohort of 381 pediatric morphea, supports the notion of ANA positivity corresponding to deeper tissue involvement (14). Using IF on Hep-2 cells, standard laboratory evaluation and cut-off, the Childhood Arthritis and Rheumatology Research Alliance (CARRA) cohort demonstrated 48% ANA positivity with a significant association of non-cutaneous disease damage with ANA, specifically for the deeper tissues of the extremity with remaining joint contractures, muscle atrophy, and extremity shortening (Supplementary Table 1) (14). In addition to the depth of the disease into adjacent connective tissue, ANA positivity has also been associated with cutaneous disease extent. The MAC cohort based in Texas (USA) and the National Registry of Childhood Onset Scleroderma (NRCOS) based in Pittsburgh (USA) both found that the extent of this skin disease (body surface area, number of lesions, number of areas affected, and mRSS) to be significantly associated with ANA positivity (19, 27). Similar to CARRA, the MAC cohort was tested using IF on Hep-2 cells, internal laboratory quality control, and healthy controls. Interpretation of the results has been standardized using a serum dilution series on the cells, and automated data processing to exclude the interlaboratory deviation.

In a prospective clinical cohort, the NRCOS, with full longitudinal data available from 77 morphea patients and an average follow-up of 5 years, a positive ANA at the baseline visit increased the odds of relapse (after obtaining remission) by a factor of 4.8 (95% CI [1.37–17.2]) (28). The same group more recently studied ANA positivity and more classic SSc-related autoantibodies in the NRCOS cohort (Supplementary Table 1). Utilizing an ANA cut-off of 1:160 via indirect immunofluorescence (IF) on Hep2 cells, 50% (35/69) pediatric morphea patients had ANA positivity, with one-third having 1:160 titer and decreasing percentages as the titer level increased, but as many as 11% had a titer of 1:1520. The most common pattern was speckled (50%), followed by homogenous, nucleolar, and centrosome (29). A panel of SSc-associated autoantibodies, including anti-topoisomerase (Scl-70), anti-centromere, U3RNP, and PM-Scl, was evaluated on all 69 subjects and were all found to be positive for 6–16% of the morphea subjects. The FIDIS™ Connective Profile SSc line immunoassay (LIA) (Euroimmun, Germany) and MagPix® (Luminex™) addressable laser bead immunoassay (ALBIA) were used to analyse morphea subject sera positivity in relation to age and sex matched healthy pediatric controls [cut-off two standard deviations (SDs) above the mean]. In general, when positive, these SSc-associated autoantibodies did not signify the diagnosis of SSc or the internal organ manifestations typically associated with SSc but correlated with morphea disease severity, such as joint contractures, musculoskeletal involvement, and skin symptoms of tingling, pain, and skin thickness (29). This supports the hypothesis that morphea (localized scleroderma) is “not just a skin disease” but that it is truly an autoimmune disease with circulating autoantibodies, associated with deeper tissue disease and more extensive connective tissue disease (28, 29).

The significant correlation of ANA and associated extrable nuclear autoantibodies with the depth of tissue involvement, extracutanous manifestations and potential for relapse in morphea, places ANA as a potential biomarker for morphea disease stratification and management, either individually or as a composite indictor with clinical variables, such as the mLoSSI, and other immune markers, such as cytokines of interest in the field, CXCL9 and CXCL10 (30, 31). CARRA investigators support ANA positivity in the prediction of muscle involvement, as is was associated with muscle atrophy, joint contracture and/or limb shortening, along with elevated muscle enzymes, CPK and aldolase, in a large North American cohort (32). A positive ANA at the baseline clinical visit for a morphea patient should prompt the clinician to closely monitor (1) the depth of the lesion, which may include further evaluation with MRI of the fascia, muscle, tendon and joint, especially the linear and generalized plaque morphea subtypes (33, 34), and (2) clinical signs of disease relapse, both during quiescent disease while on treatment and after systemic treatment course is completed (28, 35). The design of clinical trials for morphea are currently under discussion, with inclusion of ANA and other autoantibodies as exploratory biomarkers.

### Anti-histone and ss-DNA Antibodies in Morphea

**Figure 3:**
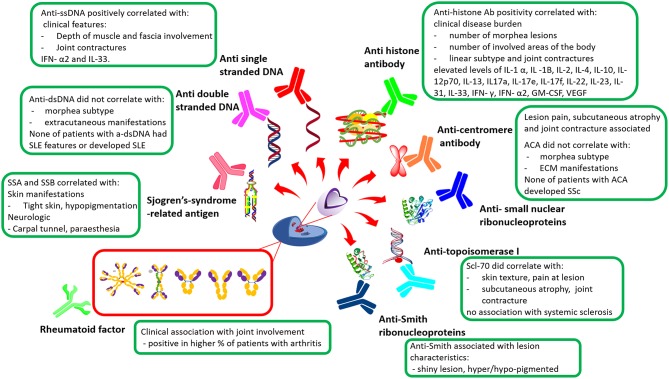
General overview of morphea autoantibodies in relation to clinical features. Anti-ssDNA, Anti-single stranded DNA; IFNx, Interferon; ILx, Interleukin; SSA, Sjogren's-syndrome-related antigen A; SSB, Sjogren's-syndrome-related antigen B; Scl-70, Topoisomerase I; ACA, Anti-centromere antibody; ECM, Extracutaneous manifestations; SSc, Systemic Sclerosis; SLE, Systemic lupus erythematosus; GM-CSF, Granulocyte-macrophage colony-stimulating factor; VEGF, Vascular endothelial growth factor; Ab, Antibody (9, 10, 13, 18, 19, 27–30, 37, 38).

In an original report of a Japanese cohort studied in the 1990s (36), anti-histone antibodies (AHAs) were positive in 47% (23/49) of the cohort. This was studied by immunoblotting and ELISA, using manual data processing, internal quality control, and a titration curve from certified tests for quantification purposes. AHA titers correlated positively with the number of lesions, larger distribution of lesions, and muscle involvement. More recent large cohort analyses in North America and Europe have also studied AHA positivity in morphea and have found similar associations (Figure 3; Supplementary Table 2). A North American nested case-control study, including both pediatric and adult morphea subjects (*n* = 187) and age, sex, and race-matched controls (*n* = 149) who were all tested for AHA via ELISA, found a significant difference in AHA positivity between morphea subjects and controls (12 vs. 2%, *p* < 0.001) (19). AHA positivity was more frequently present in the linear morphea subtype compared to the generalized plaque morphea (18 vs. 7%, *p* = 0.04) (19). The AHA may indeed associate with the linear morphea subtype, as the other large North American cohort, the NRCOS, consisting of 60% patients with linear disease subtype, reports a relatively high AHA positivity (32–39%) in a longitudinal study of subjects (30, 38). Consistent with original findings in Japan (36), in this North American cohort, AHA correlated with general disease burden, such as number of lesions and number of sites with skin involvement, especially if ≥ two cutaneous sites (as dictated by the LoSCAT score) were affected. AHA levels also correlated with the depth of the lesion, reflected by the presence of joint contractures (27, 30, 38). Although the AHA seemed to predict severity, it was not found to be a predictor of disease relapse prospectively in the NRCOS cohort (28).

Interestingly, in NRCOS, the AHA levels did have some immunological correlation to T-helper (T_H_) cell-associated cytokines evaluated by Luminex, including T_H_1-associated cytokines (IL-12p70, IFN- γ, IL-2), T_H_2-associated cytokines (Il-4, IL-13), and T_H_17 associated cytokines (IL-17a, IL-17e, IL-17f, IL-d22, IL-23) (37). The Luminex assay has been standardized using age-matched healthy controls and internal laboratory quality control. Sensitivity down to a couple of molecules/uL samples is a great advantage of a Luminex detection methodology; in addition, all data have been processed automatically. Immune activation, reflected by cutaneous disease activity, was noted in this cohort's earlier reports, with a subset of patients having longitudinal changes in their AHA levels corresponding to changes in the disease activity status (38).

Around the same time the AHA was being described in morphea, the research group in Pittsburgh identified an antibody to DNA in morphea. Originally it was thought to be directed to double-stranded (ds) DNA, but an investigation with the *Crithidia lucillae* IF assay determined negative antibodies to double-stranded DNA (a-dsDNA), but a positive anti-single stranded DNA (a-ssDNA) antibodies (27). Herein, internal laboratory control and a non-matched healthy cohorts were applied, and the data was processed automatically. Further, the study of 39 patients in this cohort found that 51% of the morphea subjects had positive anti-ssDNA, with a positive correlation between anti-ssDNA antibody and joint contracture or active disease with a duration of longer than 2 years (27) (Supplementary Table 2). These general findings were validated in a Japanese cohort, led by Takehara and Sato, who found 50% morphea subjects with a positive anti-ssDNA, which correlated with deeper involvement of the muscle and fascia (39). Anti-ssDNA levels also correlated positively with the disease activity in a subgroup of patients (39). Later studies in the childhood-onset Pittsburgh cohort, NRCOS, continued to find anti-ssDNA positivity in ~30–44% of their morphea subjects, associated with more extensive skin involvement, active disease, and inflammatory cytokines IFN-α2 and IL-33, signifying immunologically active disease (30, 38). Noteworthy, anti-ssDNA positivity did not predict relapse in this cohort followed longitudinally (28).

Double positivity for AHA and anti-ssDNA was associated with a more severe morphea phenotype with deep tissue involvement and joint contracture, especially in childhood-onset, but also adult onset (38). In contrast, the other North American cohort, MAC, found a much lower positivity of anti-ssDNA, finding 6–8% positivity among combined adult and pediatric-onset morphea, which was not significantly different from their control cohort (7%) (19, 40). It is unclear why there is a degree of difference, potentially due to the different testing methods (41), ELISA compared to IF. IF is believed to be more accurate. IF also has an automated data processing vs. the often manually performed ELISA.

In the dermatology-based United States cohort, the MAC cohort, patients with a double positivity for ss-DNA and AHA or ANA, indicated more severe morphea including functional limitations in linear disease (anti-ssDNA, *p* = 0.005; and AHA, *p* = 0.006), extensive body surface area involvement (anti-ssDNA, *p* = 0.01; and ANA, *p* = 0.005), and higher skin damage (ANA, *p* = 0.004) (19).

In concert with the ANA's ability to reflect deeper disease, such as fascia, muscle, and joint involvement, with resultant joint contractures, AHA and ss-DNA positivity share this relationship and could guide the clinician's management. This would include a more complete examination of the underlying connective tissue with a thorough joint examination and the use of available adjunct measures to monitor the depth and activity status of morphea in deeper tissues, such as MRI and Ultrasound (33, 34). A combination of positive antibodies between ANA, AHA, and ss-DNA should signify to the clinician a more severe immunophenotype with potential progression to joint contracture, which may influence decisions to initiate systemic medications and the length of treatment. Unlike ANA positivity at the baseline assessment, AHA and anti-ssDNA antibodies do not appear to influence disease relapse to assist in assessment of recurrence.

### Systemic Sclerosis and Systemic Lupus Erythematosus Autoantibodies in Morphea

Antibodies against the traditional extractable nuclear antigens (ENAs), such as anti-dsDNA, SSA/B, Smith/RNP, anti-Scl-70, and anti-centromere Abs, are seldom in morphea, with general percentages of positivity ranging from 1 to 15% internationally in both pediatric and adult onset morphea [Supplementary Table 3; (9, 10, 13, 18, 29)]. These serum autoantibodies were obtained clinically, and abnormal values were referenced to the normal range of laboratory parameters of each of the participating centers. It is important to note that clinical development of the connective tissue diseases typically associated with these ENAs, such as SLE, Sjogrens' or SSc, was not documented in any of these cohort studies (Supplementary Table 3). Only a few studies investigated clinical associations of morphea patients with these ENAs. The international pediatric cohort compared morphea subtype and presence of extracutaneous manifestation with the presence of ds-DNA, Scl-70, and centromere antibodies and did not find any associations (13, 18). However, in the prospectively collected NRCOS cohort, when comparing detailed clinical variables of morphea, such as lesion characteristics of thickness, subcutaneous atrophy, and patient symptoms, such as pain at site of the lesion, more correlations were detected with these ENAs (Supplementary Table 3) (29).

### Rheumatoid Factor

Rheumatoid factor (RF), which is typically associated with adult rheumatoid arthritis, was positive in 3–16% of morphea patients in the reported cohorts internationally (Supplementary Table 3). These serum RF values were obtained clinically, and abnormal values were referenced to the normal range of laboratory parameters of each of the participating centers. The largest cohort tested were childhood-onset patients (*n* = 464). Of this cohort, 16% of subjects were RF positive which correlated positively with arthritis and musculoskeletal manifestations (18), providing evidence for increased clinical monitoring of the joints of positive RF patients (42–44).

## Role of Autoantibodies in the Pathogenesis of Morphea

There are several recent studies aimed at an improved understanding of the basic biology of morphea. A better understanding of the underlying mechanisms in morphea, especially during the active inflammatory phase, would lead to more direct and efficient therapies (45). According to Jacobe et al. specific HLA class I and class II alleles are associated with generalized and linear subtypes of morphea (40). Notably, the morphea-associated alleles are different from those found in SSc, suggesting that morphea is immunogenetically distinct. Risk alleles in morphea are also associated with conditions such as rheumatoid arthritis (RA) and other autoimmune conditions (40). The role of HLA products in regulating interactions of immune cells is well-known (46), and therefore, the specific HLA profile of morphea could lead to B cells producing certain cytokines and autoantibodies contributing to disease progression (40, 47).

**Figure 4:**
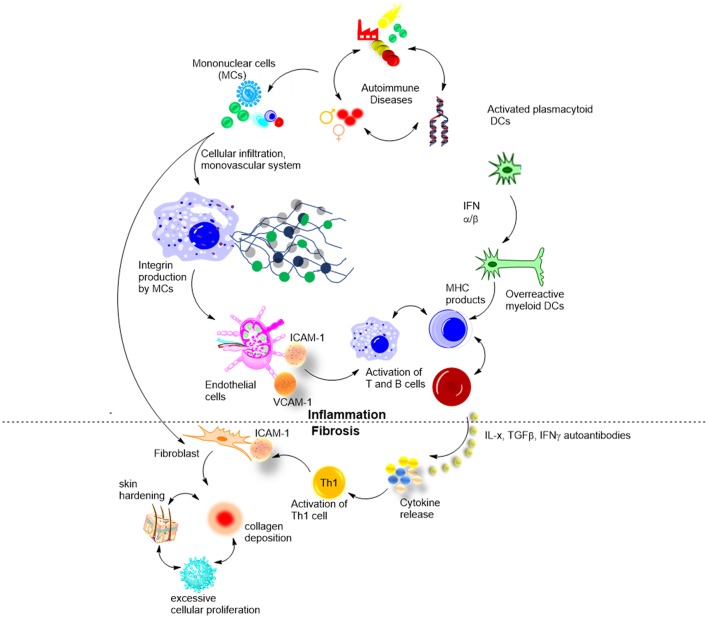
Biological mechanisms involved in morphea, and suggested roles of cytokines and autoantibodies.

Cytokine and autoantibody profiles and their relationship to clinical features in morphea have been described. It is believed that the imbalance between Th1/Th2/Th17 cell subsets drives inflammation in the early stages of disease (Th1 and Th17 predominant) and fibrosis in the later stages of morphea (Th2 predominant) (47). As in SSc, T-helper (Th) cells and their associated cytokines have been suggested to contribute significantly to the pathophysiology of the disease. This was confirmed by the presence of cytokines from Th cells in the sera and tissues of patients (Figure 4) (37).

Kurzinski and Torok analyzed the available experimental data on cytokines in morphea and compared them to available clinical disease severity and activity features (37). They confirmed that cytokines of Th1, Th2, and Th17 cell responses likely contribute to the pathogenesis of both SSc and morphea. Prior studies support the theory of Th1/Th2 imbalance in SSc propagating disease in a Th2 manner, leading to skin fibrosis and damage. Early presence of the pro-inflammatory cytokines IL-2 and IL-6 inducing Th17 in morphea patients suggests that an early increase of Th1 cytokines mediates the active, inflammatory phase of the disease (which was further supported by their later work) (30), and the subsequent inflammation and transition to the fibrotic damage phase is achieved via Th17. This is further supported by the elevated Th17 effector cytokines 2–4 years after the initial onset of disease. In turn, Th2 effector cytokines shown to be present at elevated levels in morphea promote the development of tissue damage and fibrosis later in the course of the disease (37).

Badea et al. reviewed step-wise development of morphea (Figure 4) (48). In early inflammatory stages, environmental stimuli and genetic factors activate mononuclear cells that in turn induce perivascular infiltration of the skin. At this stage, ANA, cytokines, and other soluble cell-adhesion molecules are elevated, confirming on-going immune activation. At stage 2, functional and structural changes occurred to the microvascular system, and adhesion molecules including ICAM-1 and VCAM-1, were upregulated in response to various cytokines and cell mediators (IFN-γ, IL-1, TNFs). The third stage is the least understood, and it is believed to involve a large release of cytokines by the lymphocytes. IL-4, IL-6, and IL-8 are among those overproduced cytokines. Stage four involves hardening of the skin from excessive cellular proliferation and deposition of collagen and other extracellular matrix components. This stage of LS progression has the most deleterious effects and is believed to be driven by excess IL-4 and TGF-β (48). This general pathogenesis model has been confirmed by *in vitro* studies in cohorts of morphea patients at different disease stages, and by knock-out mouse models (Tsk). Using novel antigens *in vivo* could bring new insights into pathogenesis. Moreover, detailed studies of T and B cell populations in morphea vs. controls, e.g., SSc and psoriasis, are lacking (49).

Osmola–Mankowska et al. added new knowledge to the existing picture of morphea pathogenesis, by proposing the underseen role of dendritic cells (DCs) in it (Figure 4) (50). In brief, an unknown natural ligand activates DC to produce IFN-α and IFN-β that in turn activate myeloid DCs (mDCs). mDCs activate autoreactive B and T cells via MHC molecules, which then leads to skin autoimmunity and morphea in particular. The hypothesis has been confirmed by both *in vitro* and *in vivo* studies and is an exciting new knowledge with potential therapeutic outcomes (50). T-cell subtyping has been successfully used to identify *in vivo* the regulatory pathways associated with morphea, specifically, confirming that apoptotic bodies bearing CD8, activate CD205 of DCs. This knowledge allows the application of already existing therapies to morphea, such as pDC regulating SLE drug candidates (Anti-BDCA-2 Abs and nanobodies).

Verification of the role of autoantibodies in morphea is complicated by only a few available animal models. Marangoni et al. reviewed available models of scleroderma (51) and provided details on nine key mouse models available today, created by exogenous administration of fibrosis-inducing agents (bleomycin, Ad-TGFβ1^223/225^), and by genetic manipulation (e.g., Tsk-2 and Fbn-1 mutants). The high diversity of skin disease symptoms complicates creating a reliable model for morphea. The authors suggest using genome-wide expression analysis to match the animal models to the appropriate subtypes of human clinical disease. Additionally, there is a high deviation in methodology for inducing scleroderma pathogenesis with bleomycin and Ad-TGFβ1^223/225^ that remains unresolved. In spite of this, mouse models of cutaneous fibrosis are proven to be useful to study underlying mechanisms of the disease and its linkage to other conditions (51).

Several other mouse models of skin fibrosis were reported as useful to study the onset of the disease (52). Tsk1 and Tsk2 models are developed through mutations in fibrillin gene *FBN1* leading to fibrinogenesis. It is known that fibrillin-1 is a component of connective tissue microfibrils and is important in correcting elastic fiber assembly. Second, fibrillin-1 can partially control TGF-β availability via confirmed interactions with TGF-β binding proteins 1 and 4. Genetic analysis of 6-week-old Tsk mutant mice indicated morphea related effects: upregulated collagen synthesis, increased bone morphogenic protein, and connective tissue growth factor. Additionally, Wnt signaling proteins that interfere with TGF-β are overproduced. With regard to serology, 88% of Tsk mutant models are ANA positive, and also contain AHA, anti-Scl-70, and anti-RNApol II antibodies (52). This makes Tsk models potent tools for studying autoantibodies in morphea. A limitation is the fact that Tsk model lacks inflammation histologically, therefore it reflects SSc skin condition more than morphea skin. Bleomycin-induced mouse is an alternative to Tsk for studies of morphea which develops a skin inflammation and a set of autoantibodies (51, 52).

## Emerging Autoantibody Biomarkers in Morphea

Autoantibodies to the dense fine speckled 70 kDa antigen (anti-DFS70) is a hot topic in autoimmune research and diagnostics at the moment. Anti-DFS70 are reported to be more common in individuals who do not have an antinuclear antibody (ANA)-associated rheumatic disease (AARD) than in patients with AARD. So far, the frequency of anti-DFS70 antibodies has been thoroughly studied in adults but not in pediatric populations. The primary objective of a recent observational study was to determine the frequency of anti-DFS70 in pediatric AARD including morphea, and reference cohorts (53). Sera from 743 children with AARD and related conditions and 345 samples from controls [healthy children and suspected for AARD] were tested for anti-DFS70 antibodies. Using a novel chemiluminescence assay, anti-DFS70 antibodies were elevated in 2.1% of healthy children and 4.5% of sera from ANA positive pediatric samples. Information on standardization procedure was missing for anti-DFS70 that might lead to deviation from other cohorts. Notably, in line with previous studies which suggest an overlap of morphea with other diseases, the frequency of anti-DFS70 was highest in juvenile morphea (13.8%), along with juvenile dermatomyositis (18.2%), childhood SLE (5.7%), and juvenile idiopathic arthritis (2.5%) (53).

**Figure 5:**
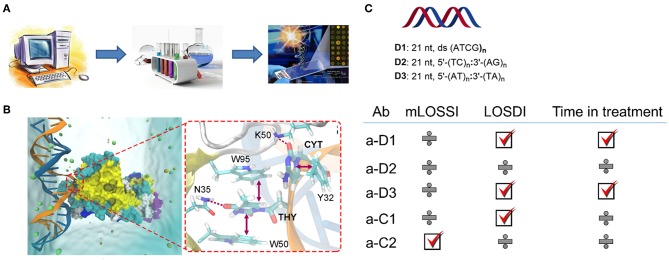
Synthetic DNA, RNA, and LNA antigens in morphea (56). **(A)** A general approach to the design including computation, library screening, and ELISA; **(B)** representative molecular dynamics result for dsDNA antigen and autoantibody showing key interactions contributing to the binding; **(C)** Representative antigens selected for the study of the pediatric morphea cohort, and correlations of ELISA results with modified localized scleroderma skin severity index (mLOSSI), index of disease damage (LOSDI), and time in treatment. C1 and C2 were commercial controls used in the study, calf thymus DNA, and G-quadruplex human DNA.

In our recent study, we developed a series of synthetic oligonucleotides (54, 55) that allowed us to investigate the details on the antigen recognition by autoimmune antibodies in pediatric morphea (Figure 5) (56). In this work, we hypothesized that having a sequence-controlled rationally designed DNA, RNA, and locked nucleic acid (LNA) antigen might provide new insights into sequence-specific binding of anti-ssDNA and anti-dsDNA. Typically used in nucleic acid diagnostics and gene therapy (57, 58) synthetic oligo- and poly-nucleotides are poorly explored in immunology. However, these molecules have major advantages of high purity, controlled chemical content and a possibility to incorporate functional tags (54, 55). To design new antigens which were 21–63 nucleotide long oligo- and polymers, we successfully combined computation and library screening (56, 59). The study has been benchmarked to SLE (*n* = 30) and healthy controls (*n* = 80); standardization has been done using internal laboratory control and external calibrators provided by Odense University Hospital, Denmark, and Stanford University Hospital, CA, USA. Besides dramatically improving the analytical specificity of the assay, our data suggest a potential link between antibodies to DNA and the disease state in morphea. Moreover, introducing chemical modification (LNA) into antigens completely changed the binding of corresponding antibodies and their clinical relevance. The strongest observed effect was seen for the localized scleroderma skin damage index (LoSDI) on the IgG antibodies to TC dinucleotide-rich dsDNA (*p* < 0.001) (56). Synthetic DNA and analogs are therefore a new promising class of antigens that could bring light into sequence specificity of anti-DNA antibodies in morphea and related diseases. Lack of confirmation for antigen-antibody reactivity in a relevant morphea animal model is a limitation of this work.

Looking for gene association of autoimmune diseases including morphea is a rapidly developing research field with high potential to provide new insights into pathogenesis and new biomarkers. Earlier, Torok et al. reported an up-regulated IFN-related gene CXCL10 in pediatric morphea subjects (30). Simultaneously, O'Brien et al. investigated transcriptional and cytokine profiles in 87 adult morphea subjects and 26 healthy controls (31). This study identified a disease severity association for CXCL9, which was present at increased levels in active morphea subjects (37%) along with Th1 cell cytokines (57%). Related gene CXCL10 was upregulated in 44% morphea subjects but did not correlate with disease severity (31). CXCL9/10 studies also led to a new hypothesis on the onset of morphea. As a result of cutaneous overproduction of IFN-γ by cutaneous macrophages, Th1 imbalance in the skin could be a contributing factor to disease progression. Thus, local skin autoimmunity could be the driver of the disease in contrast to the systemic dysregulation in SSc (31). In (31), a higher number of healthy controls and additional disease controls could be included to support this study.

## Conclusions

As this review summarizes, morphea (localized scleroderma) is a complex disease with a diverse profile of clinical manifestations and still unresolved issues with serological diagnosis, clear knowledge on pathogenesis, and missing of effective management routes. In this scoping review, we selected studies based on the number of enrolled patients with autoantibody testing (≥50 patients), relevance to studies of morphea pathogenesis, and reported emerging animal models and biomarkers that could lead to improved personalized morphea management. Up to 50% of patients in cohorts described herein have elevated levels of three main autoantibodies: ANA, AHA, and anti-ssDNA, whereas other autoantibodies are observed at frequencies below 10%.

These autoantibodies associate with more severe disease, including lesion depth and spread, associating with more severe morphea subtypes and extracutaneous manifestations. The presence of two or more of these autoantibodies appears to have a cumulative effect on correlation to disease affecting the deep muscle, fasica and tendons, resulting in joint contracture and limited mobility. Rheumatoid factor also appears to be a strong indicator of deeper tissue disease with arthritis association. Taken together, patients with ANA, AHA, ss-DNA, or RF positivity in isolation, or more importantly in combination, are at higher risk for muscle and joint morbidity. Extra care should be taken to ensure these patients have a detailed musculoskeletal examination (in addition to a thorough cutaneous assessment), and if possible, consider imaging deeper tissue to monitor the depth of moprhea involvement and the disease activity status (i.e.,–edema of the fascia on MRI).

Furthermore, morphea patients with multiple autoantibody positivity might have a more robust B cell activation, promoting such antibodies, and may benefit from anti-CD20 therapy, such as Rituximab, currently available for rheumatoid arthritis and other connective tissue disease indications (60). Only a few case reports are available evaluating Rituximab in morphea, but do show successful response (including improvement of deeper tissue via MRI evaluation) in previous recalcitrant disease (61, 62). Another potential therapeutic target in morphea is between the T cell and antigen presenting cell (DC or B-cell) communication, via abatacept, a fusion protein that inhibits CD80/86 interaction with CD28. This can dampen B cell:T-cell interactions and T-cell functional activation, which in turn would lead to less inflammatory driven fibrosis and potentially less auto-antibody production. A few cases reports have described successful treatment of morphea with abatacept, especially widespread disease or deeper disease (monitored by MRI in some) (63–65). Further clinical studies in trial format are warranted to further understand the utility of these biologic agents in treating morphea.

Pathogenesis of morphea remains being a black box, although there are several recent works that bring new knowledge to the field. The reported association with HLA I and HLA II alleles defined by Jacobe et al., could be a breakthrough in the genotype based diagnosis, subtyping and research on morphea (40). Genomic association with CXCL9 is another exciting direction toward these goals (31). Recently, a role of DCs in the critical step of morphea development has been recently confirmed (50). Tsk animal models show high levels of autoantibodies and similar fibrotic skin features to human morphea, making Tsk mouse a valuable model for further studies, although the inflammatory state is somewhat limited in the Tsk mouse (51, 52). As more information is gained about the underlying mechanisms of morphea, diagnosis and treatments can become more accurate and better personalized. Importantly, improving scleroderma patients' early diagnosis before extracutaneous manifestations occur should improve patients long term recovery (45, 66).

Furthermore, beneficial autoantibodies having protective effects against the development of immune-mediated diseases and conventional antibodies depend on the activation of T and B lymphocytes by antigen presenting DCs and share common ontogeny (67, 68). Thus, it is suspected that the disturbance in the homeostasis of autoantibodies can trigger autoimmune diseases. Cabral-Marques reported that dysregulation of autoantibody-targeting G protein-coupled receptors (GPCRs) can trigger the development of rheumatic diseases including rheumatoid arthritis and SSc. Sera from 84 patients with SSc and 491 healthy controls were tested for anti- GPCRs antibodies. Using commercial solid-phase sandwich ELISA, anti-GPCRs concentration were either elevated or decreased in sera of SSc and rheumatoid arthritis in contrast to healthy samples. Hence, the discovery of anti-GPCR autoantibodies in pathogenesis of rheumatic diseases opens up opportunities for new investigations in autoimmune diseases including morphea and SSc (68).

The potential impact of synthetic biology and computational chemistry in improving the efficacy and specificity of existing antigens might present an exciting new approach in the management of morphea. For example, a combined computation and library screening provided with a new TC rich dsDNA antigen that allows for detecting autoantibodies associated with skin damage index.

Increased evidence shows that environmental factors and other diseases may have an impact on morphea. Emerging biomarkers including anti-DFS70 and anti-LNA/DNA, aim at detecting these associations, which opens up new pathways for managing difficult and rare cases of morphea (69, 70).

## Author Contributions

SK and KA: prepared initial draft. All authors collected and analyzed data and research papers, prepared graphics, proof read the paper, and prepared the final version.

### Conflict of Interest Statement

The authors declare that the research was conducted in the absence of any commercial or financial relationships that could be construed as a potential conflict of interest.

## References

[1] Cotton CV Spencer LG New RP Cooper RG. The utility of comprehensive autoantibody testing to differentiate connective tissue disease associated and idiopathic interstitial lung disease subgroup cases. Rheumatology. (2017) 56:1264–71. 10.1093/rheumatology/kew320 28339528 PMC5850114

[2] Kontny E Lewandowska-Poluch A Chmielinska M Olesinska M. Subgroups of Sjögren's syndrome patients categorised by serological profiles: clinical and immunological characteristics. Reumatologia. (2018) 56:346–53. 10.5114/reum.2018.80711 30647480 PMC6330679

[3] Navallas M Inarejos Clemente EJ Iglesias E Rebollo-Polo M Antón J Navarro OM. Connective tissue disorders in childhood: are they all the same? Radiographics. (2019) 39:229–50. 10.1148/rg.2019180078 30620697

[4] Steen VD Medsger TA. Improvement in skin thickening in systemic sclerosis associated with improved survival. Arthritis Rheum. (2001) 44:2828–35. 10.1002/1529-0131(200112)44:12<2828::AID-ART470>3.0.CO;2-U 11762943

[5] Bernatsky S Joseph L Pineau CA Belisle P Hudson M Clarke AE. Scleroderma prevalence: demographic variations in a population-based sample. Arthritis Rheum. (2009) 61:400–4. 10.1002/art.24339 19248123

[6] Laxer RM Zulian F. Localized scleroderma. Curr Opin Rheumatol. (2006) 18:606–13. 10.1097/01.bor.0000245727.40630.c3 17053506

[7] Ardalan K Zigler CK Torok KS. Predictors of longitudinal quality of life in juvenile localized scleroderma. Arthritis Care Res. (2017) 69:1082–7. 10.1002/acr.23101 27696700 PMC5376376

[8] Condie D Grabell D Jacobe H. Comparison of outcomes in adults with pediatric-onset morphea and those with adult-onset morphea: a cross-sectional study from the morphea in adults and children cohort. Arthritis Rheumatol. (2014) 66:3496–504. 10.1002/art.38853 25156342 PMC4245331

[9] Kreuter A Wischnewski J Terras S Altmeyer P Stücker M Gambichler T. Coexistence of lichen sclerosus and morphea: a retrospective analysis of 472 patients with localized scleroderma from a German tertiary referral center. J Am Acad Dermatol. (2012) 67:1157–62. 10.1016/j.jaad.2012.04.003 22533994

[10] Leitenberger JJ Cayce RL Haley RW Adams-Huet B Bergstresser PR Jacobe HT. Distinct autoimmune syndromes in morphea: a review of 245 adult and pediatric cases. Arch Dermatol. (2009) 145:545–50. 10.1001/archdermatol.2009.79 19451498 PMC2938024

[11] Li SC. Scleroderma in children and adolescents: localized scleroderma and systemic sclerosis. Pediatr Clin North Am. (2018) 65:757–81. 10.1016/j.pcl.2018.04.002 30031497

[12] Li SC Li X Pope E Stewart K Higgins GC Rabinovich CE. New features for measuring disease activity in pediatric localized scleroderma. J Rheumatol. (2018) 45:1680–8. 10.3899/jrheum.171381 30219769

[13] Zulian F Vallongo C Woo P Russo R Ruperto N Harper J Localized scleroderma in childhood is not just a skin disease. Arthritis Rheum. (2005) 52:2873–81. 10.1002/art.21264 16142730

[14] Description of the Juvenile Localized Scleroderma Subgroup of the Childhood Arthritis and Rheumatology Research Alliance (CARRA) Registry—ACR Meeting Abstracts. Available online at: https://acrabstracts.org/abstract/description-of-the-juvenile-localized-scleroderma-subgroup-of-the-childhood-arthritis-and-rheumatology-research-alliance-carra-registry/ (accessed May 25, 2019). 10.1002/acr2.1019 PMC6858014 31777788

[15] Kister I Inglese M Laxer RM Herbert J. Neurologic manifestations of localized scleroderma: a case report and literature review. Neurology. (2008) 71:1538–45. 10.1212/01.wnl.0000334474.88923.e3 18981376

[16] Moko SB Mistry Y Blandin de Chalain TM. Parry-Romberg syndrome: intracranial MRI appearances. J Craniomaxillofac Surg. (2003) 31:321–4. 10.1016/S1010-5182(03)00028-3 14563334

[17] Arkachaisri T Vilaiyuk S Torok KS Medsger TA. Development and initial validation of the localized scleroderma skin damage index and physician global assessment of disease damage: a proof-of-concept study. Rheumatology. (2010) 49:373–81. 10.1093/rheumatology/kep361 20008472 PMC3498950

[18] Zulian F Athreya BH Laxer R Nelson AM Feitosa de Oliveira SK Punaro MG. Juvenile localized scleroderma: clinical and epidemiological features in 750 children. An international study. Rheumatology. (2006) 45:614–20. 10.1093/rheumatology/kei251 16368732

[19] Dharamsi JW Victor S Aguwa N Ahn C Arnett F Mayes MD. Morphea in adults and children cohort III: nested case-control study–the clinical significance of autoantibodies in morphea. JAMA Dermatol. (2013) 149:1159–65. 10.1001/jamadermatol.2013.4207 23925398 PMC4153681

[20] Freeman MF Tukey JW Transformations related to the angular and the square root. Ann Math Statist. (1950) 21:607–11. 10.1214/aoms/1177729756

[21] Miller JJ The inverse of the Freeman – Tukey double arcsine transformation. Am Stat. (1978) 32:138 10.1080/00031305.1978.10479283

[22] DerSimonian R Laird N. Meta-analysis in clinical trials. Control Clin Trials. (1986) 7:177–88. 10.1016/0197-2456(86)90046-2 3802833

[23] http://www.iecs.org.ar/cochrane/guias/Handbook_4-2-2.pdf.

[24] Higgins JPT Thompson SG. Quantifying heterogeneity in a meta-analysis. Stat Med. (2002) 21:1539–58. 10.1002/sim.1186 12111919

[25] Lau J Ioannidis JP Schmid CH. Quantitative synthesis in systematic reviews. Ann Intern Med. (1997) 127:820–6. 10.7326/0003-4819-127-9-199711010-00008 9382404

[26] Dear KBG Begg CB An approach for assessing publication bias prior to performing a meta-analysis. Stat Sci. (1992) 7:237–45. 10.1214/ss/1177011363

[27] Falanga V Medsger TA Reichlin M Rodnan GP. Linear scleroderma. Clinical spectrum, prognosis, and laboratory abnormalities. Ann Intern Med. (1986) 104:849–57. 10.7326/0003-4819-104-6-849 3486617

[28] Kurzinski KL Zigler CK Torok KS. Prediction of disease relapse in a cohort of paediatric patients with localized scleroderma. Br J Dermatol. (2019) 180:1183–9. 10.1111/bjd.17312 30315656 PMC6462250

[29] Autoantibody Testing in Pediatric Localized Scleroderma (LS) - ACR Meeting Abstracts. Available online at: https://acrabstracts.org/abstract/autoantibody-testing-in-pediatric-localized-scleroderma-ls/ (accessed March 27, 2019)

[30] Torok KS Kurzinski K Kelsey C Yabes J Magee K Vallejo AN. Peripheral blood cytokine and chemokine profiles in juvenile localized scleroderma: T-helper cell-associated cytokine profiles. Semin Arthritis Rheum. (2015) 45:284–93. 10.1016/j.semarthrit.2015.06.006 26254121 PMC4656125

[31] O'Brien JC Rainwater YB Malviya N Cyrus N Auer-Hackenberg L Hynan LS. Transcriptional and cytokine profiles identify CXCL9 as a biomarker of disease activity in morphea. J Invest Dermatol. (2017) 137:1663–70. 10.1016/j.jid.2017.04.008 28450066 PMC6217816

[32] Wu EY Li SC Torok KS Virkud YV Fuhlbrigge RC Rabinovich CE Baseline description of the juvenile localized scleroderma subgroup from the childhood arthritis and rheumatology research alliance legacy registry. ACR Open Rheumatol. (2019) 1:119–24. 10.1002/acr2.1019 PMC6858014 31777788

[33] Eutsler EP Horton DB Epelman M Finkel T Averill LW. Musculoskeletal MRI findings of juvenile localized scleroderma. Pediatr Radiol. (2017) 47:442–9. 10.1007/s00247-016-3765-x 28091699

[34] Schanz S Henes J Ulmer A Kötter I Fierlbeck G Claussen CD. Response evaluation of musculoskeletal involvement in patients with deep morphea treated with methotrexate and prednisolone: a combined MRI and clinical approach. Am J Roentgenol. (2013) 200:W376–82. 10.2214/AJR.12.9335 23521481

[35] Mirsky L Chakkittakandiyil A Laxer RM O'Brien C Pope E Relapse after systemic treatment in pediatric morphoea. Br J Dermatol. (2012) 166:443–5. 10.1111/j.1365-2133.2011.10535.x 21793814

[36] Sato S Ihn H Soma Y Igarashi A Tamaki T Kikuchi K. Antihistone antibodies in patients with localized scleroderma. Arthritis Rheum. (1993) 36:1137–41. 10.1002/art.1780360815 8343189

[37] Kurzinski K Torok KS. Cytokine profiles in localized scleroderma and relationship to clinical features. Cytokine. (2011) 55:157–64. 10.1016/j.cyto.2011.04.001 21536453 PMC3632442

[38] Arkachaisri T Fertig N Pino S Medsger TA. Serum autoantibodies and their clinical associations in patients with childhood- and adult-onset linear scleroderma. A single-center study. J Rheumatol. (2008) 35:2439–44. 10.3899/jrheum.080098 19004036

[39] Takehara K Sato S. Localized scleroderma is an autoimmune disorder. Rheumatology. (2005) 44:274–9. 10.1093/rheumatology/keh487 15561734

[40] Jacobe H Ahn C Arnett FC Reveille JD. Major histocompatibility complex class I and class II alleles may confer susceptibility to or protection against morphea: findings from the Morphea in Adults and Children cohort. Arthritis Rheumatol. (2014) 66:3170–7. 10.1002/art.38814 25223600 PMC4211936

[41] Takehara K Moroi Y Nakabayashi Y Ishibashi Y. Antinuclear antibodies in localized scleroderma. Arthritis Rheum. (1983) 26:612–6. 10.1002/art.1780260506 6405756

[42] Zulian F Vallongo C Patrizi A Belloni-Fortina A Cutrone M Alessio M A long-term follow-up study of methotrexate in juvenile localized scleroderma (morphea). J Am Acad Dermatol. (2012) 67:1151–6. 10.1016/j.jaad.2012.03.036 22657157

[43] Knobler R Moinzadeh P Hunzelmann N Kreuter A Cozzio A Mouthon L. European dermatology forum S1-guideline on the diagnosis and treatment of sclerosing diseases of the skin, Part 1: localized scleroderma, systemic sclerosis and overlap syndromes. J Eur Acad Dermatol Venereol. (2017) 31:1401–24. 10.1111/jdv.14458 28792092

[44] Mimura Y Ihn H Jinnin M Asano Y Yamane K Tamaki K. Rheumatoid factor isotypes in localized scleroderma. Clin Exp Dermatol. (2005) 30:405–8. 10.1111/j.1365-2230.2005.01776.x 15953082

[45] Brady SM Shapiro L Mousa SA. Current and future direction in the management of scleroderma. Arch Dermatol Res. (2016) 308:461–71. 10.1007/s00403-016-1647-6 27139430

[46] Gough SCL Simmonds MJ. The HLA region and autoimmune disease: associations and mechanisms of action. Curr Genom. (2007) 8:453–65. 10.2174/138920207783591690 19412418 PMC2647156

[47] Hasegawa M Fujimoto M Kikuchi K Takehara K. Elevated serum levels of interleukin 4 (IL-4), IL-10, and IL-13 in patients with systemic sclerosis. J Rheumatol. (1997) 24:328–32. 10.1016/0923-1811(96)89424-2 9034992

[48] Badea I Taylor M Rosenberg A Foldvari M. Pathogenesis and therapeutic approaches for improved topical treatment in localized scleroderma and systemic sclerosis. Rheumatology. (2009) 48:213–21. 10.1093/rheumatology/ken405 19022832

[49] Caielli S Veiga DT Balasubramanian P Athale S Domic B Murat E. A CD4+ T cell population expanded in lupus blood provides B cell help through interleukin-10 and succinate. Nat Med. (2019) 25:75–81. 10.1038/s41591-018-0254-9 30478422 PMC6325012

[50] Osmola-Mankowska A Teresiak-Mikołajczak E Danczak-Pazdrowska A Kowalczyk M Zaba R Adamski Z. The role of dendritic cells and regulatory T cells in the pathogenesis of morphea. Cent Eur J Immunol. (2015) 40:103–8. 10.5114/ceji.2015.50841 26155191 PMC4472547

[51] Marangoni RG Varga J Tourtellotte WG. Animal models of scleroderma: recent progress. Curr Opin Rheumatol. (2016) 28:561–70. 10.1097/BOR.0000000000000331 27533324

[52] Smith GP Chan ESL. Molecular pathogenesis of skin fibrosis: insight from animal models. Curr Rheumatol Rep. (2010) 12:26–33. 10.1007/s11926-009-0080-7 20425530 PMC2861786

[53] Schmeling H Mahler M Levy DM Moore K Stevens AM Wick J. Autoantibodies to dense fine speckles in pediatric diseases and controls. J Rheumatol. (2015) 42:2419–26. 10.3899/jrheum.150567 26472409

[54] Astakhova IK Kumar TS Campbell MA Ustinov AV Korshun VA Wengel J. Branched DNA nanostructures efficiently stabilised and monitored by novel pyrene-perylene 2'-α-L-amino-LNA FRET pairs. Chem Commun. (2013) 49:511–3. 10.1039/C2CC37547H 23201901

[55] Astakhova IK Pasternak K Campbell MA Gupta P Wengel J. A locked nucleic acid-based nanocrawler: designed and reversible movement detected by multicolor fluorescence. J Am Chem Soc. (2013) 135:2423–6. 10.1021/ja311250w 23379691

[56] Samuelsen S Jørgensen CD Mellins ED Torok KS Astakhova K. Detection of autoimmune antibodies in localized scleroderma by synthetic oligonucleotide antigens. PLoS ONE. (2018) 13:e0195381. 10.1371/journal.pone.0195381 29641558 PMC5895021

[57] Astakhova K Toward non-enzymatic ultrasensitive identification of single nucleotide polymorphisms by optical methods. Chemosensors. (2014) 2:193–206. 10.3390/chemosensors2030193

[58] Taskova M Madsen CS Jensen KJ Hansen LH Vester B Astakhova K. Antisense oligonucleotides internally labeled with peptides show improved target recognition and stability to enzymatic degradation. Bioconjug Chem. (2017) 28:768–74. 10.1021/acs.bioconjchem.6b00567 28292178

[59] Klecka M Thybo C Macaubas C Solov'yov I Simard J Balboni IM. Autoantibody profiling in lupus patients using synthetic nucleic acids. Sci Rep. (2018) 8:5554. 10.1038/s41598-018-23910-5 29615791 PMC5883037

[60] Lafyatis R Kissin E York M Farina G Viger K Fritzler MJ. B cell depletion with rituximab in patients with diffuse cutaneous systemic sclerosis. Arthritis Rheum. (2009) 60:578–83. 10.1002/art.24249 19180481 PMC2637937

[61] Traboulsi D Kaminska EA Barr SG Hunter C Mydlarski PR. Morphea associated with primary biliary cirrhosis and Waldenstrom macroglobulinemia: response to rituximab. JAAD Case Rep. (2018) 4:784–7. 10.1016/j.jdcr.2018.04.016 30246126 PMC6141651

[62] Chimenti Stefani MS Teoli M Di A Giunta A Esposito M Perricone R. Resolution with rituximab of localized scleroderma occurring during etanercept treatment in a patient with rheumatoid arthritis. Eur J Dermatol. (2013) 23:273–4. 10.1684/ejd.2013.1929 23557629

[63] Stausbøl-Grøn B Olesen AB Deleuran B Deleuran MS. Abatacept is a promising treatment for patients with disseminated morphea profunda: presentation of two cases. Acta Derm Venereol. (2011) 91:686–8. 10.2340/00015555-1136 21901244

[64] Adeeb F Anjum S Hodnett P Kashif A Brady M Morrissey S. Early- and late-stage morphea subtypes with deep tissue involvement is treatable with Abatacept (Orencia). Semin Arthritis Rheum. (2017) 46:775–81. 10.1016/j.semarthrit.2016.08.018 27773434

[65] Fage SW Arvesen KB Olesen AB. Abatacept improves skin-score and reduces lesions in patients with localized scleroderma: a case series. Acta Derm Venereol. (2018) 98:465–6. 10.2340/00015555-2878 29313055

[66] Saracino AM Denton CP Orteu CH. The molecular pathogenesis of morphoea: from genetics to future treatment targets. Br J Dermatol. (2017) 177:34–46. 10.1111/bjd.15001 27553363

[67] Ludwig RJ Vanhoorelbeke K Leypoldt F Kaya Z Bieber K McLachlan SM. Mechanisms of autoantibody-induced pathology. Front Immunol. (2017) 8:603. 10.3389/fimmu.2017.00603 28620373 PMC5449453

[68] Cabral-Marques O Marques A Giil LM De Vito R Rademacher J Günther J. GPCR-specific autoantibody signatures are associated with physiological and pathological immune homeostasis. Nat Commun. (2018) 9:5224. 10.1038/s41467-018-07598-9 30523250 PMC6283882

[69] Cho JH Gregersen PK. Genomics and the multifactorial nature of human autoimmune disease. N Engl J Med. (2011) 365:1612–23. 10.1056/NEJMra1100030 22029983

[70] Denton CP Ong VH. Targeted therapies for systemic sclerosis. Nat Rev Rheumatol. (2013) 9:451–64. 10.1038/nrrheum.2013.46 23567456

