# Supplementary material for: Autoantibodies in Morphea: An Update
Source: Front Immunol. 2019 Jul 9;10:1487. doi: 10.3389/fimmu.2019.01487 (PMC6634257; doi:10.3389/fimmu.2019.01487)
Supplement: Supplementary file 1 [file Table_1.pdf]

Supplementary Table 1. Current antibody tests in morphea and clinical associations of their results: Antinuclear antibodies (ANA).

Target	Ref Country and Cohort	Method details	# pts in cohort<br><br>subtype	Type of cohort	# pts, type, cont-rols	Mean age (range) in years	% Fe-male	% positive (total)	Clinical association
**International**									
ANA	(13, 18)<br><br>**Cohorts:**<br>38 European;<br>12 North American;<br>11 South American;<br>8 Asian;<br>1 Australian	Retrospective collection of patients' data from PRES and PRINTO	**750 pts:**<br><br>-489 (65%) linear<br>-113 (15%) linear head<br>-194 (26%) plaque<br>-51 (7%) generalized<br>-16 (2%) deep<br><br>-514 (68%) skin involvement only<br>-157 (21%) with Extracutaneous manifestations (ECM)	Peds	750 peds	7.3 (0-16)	70.5%	42.3% (284/671)	Largest LS cohort to date with ANA measured in 671 subjects<br><br>ANA pos. not significantly different in subtypes<br><br>ANA was significantly higher in pts with ECM than those with skin involvement only
**N American**									
ANA	(14)<br><br>**CARRA LS cohort:**<br><br>USA<br>Canada	N.D.	**381 pts:**<br><br>-207 (54%) linear<br>-60 (16%) circumscribed morphea<br>-34 (9%) generalized<br>-4 (1%) eosinophilic fasciitis<br>-4 (1%) pansclerotic<br><br>-175 (46%) with 1 or more ECM	Peds	381 peds	8.2<br>(0 – 17)	76%	48% (142/296)	ANA pos. was associated with features of non-cutaneous disease damage, specifically joint contractures OR 1.79, muscle atrophy OR 2.02, and extremity shortening OR 3.19
ANA	(10)<br><br>United States<br><br>**MAC cohort**	Indirect immunofluo-rescence (Hep2 cells)<br><br>Titer > 1:160 pos.	**245 pts:**<br><br>123 adults<br>122 peds<br><br>-88 (36%) plaque<br>-63 (26%) linear<br>-37(15%) generalized<br>-3 (1%) deep<br>-33 (14%) mixed<br>-21 (8%) other	Adults and peds	123 adults<br><br>122 peds	44.7 adults<br><br>8.7 peds	84% adults<br><br>79% peds	39.3% (35/89)<br><br>8/25 (32%) in plaque<br>6/21 (28.6%) in linear<br>11/19 (57.9%) in generalized<br>7/13 (53.8%) in Mixed<br><br>53% adults (19/36)<br>30% peds (16/53)	Pts with generalized and mixed subtypes had the highest frequency.<br><br>More frequently positive in adults<br><br>This cohort demonstrated a trend toward segregation of subtype with ANA pattern (generalized with homogenous, linear and plaque with speckled and

									mixed with nucleolar pattern)
ANA	(19)<br><br>United States<br><br>**MAC cohort**	Indirect immunofluo-rescence (Hep2 cells)<br><br>Titer > 1:80 pos.	**251 pts:**<br><br>187 (74%) included in analysis	Adults and peds	110 adults<br><br>77 peds<br><br>>1800 healthy adults+ peds	45.3 adults onset<br><br>10.1 peds onset	82%	34% (63/187) total<br><br>81% (52/63) speckled pattern<br><br>57% (36 pts) had titers 1:160 or greater	ANA present with greater frequency in morphea than in nested case controls; ANA not associated with particular subtype<br><br>Found association between presence of ANAs and extensive AHA<br><br>Association between ANA pos. and higher mRSS
ANA<br><br>(anti-ssDNA)	(27)<br><br>United States<br><br>**Pittsburgh** 1962-1983	IF on both mouse-kidney and Hep2 cells	**53 pts:**<br><br>-22 (41%) linear trunk/extremity + plaque morphea<br>-19 (36%) linear extremity<br>-7 (13%) En coupe<br>and reminder 5 en coupe + M or LinE	Adults and peds onset	44 adults onset<br><br>25 peds onset	8 (1 – 67)<br><br>44 pts (83%) <25 yr onset<br><br>9 pts >25 yr onset	80%	53 (100%)<br>46% when tested on Hep2 cells<br>31% when serum was tested on mouse kidney substrate<br><br>75% homogenous<br>17% speckled<br>8% nucleolar	ANA pos. more common in pts with severe and extensive disease<br><br>All pts with positive ANA on mouse kidney, duration of disease was >2 yrs
ANA<br><br>(AHA)<br><br>(anti-ssDNA)	(38)<br><br>United States<br><br>**Pittsburgh** 1991-2005	Indirect immunofluo-rescence (Hep2 cells) and ELISA	**72 pts:**<br><br>-72 (100%) linear scleroderma<br>-12 (17%) ECDS<br>-60 (83%) Lin Extr	Adults and peds onset	32 adult onset<br><br>40 peds onset	32.9 (+/- 14.5) adult onset<br><br>8.8 (+/- 3.9) yrs peds onset	81%<br><br>84% adult (27 pts)<br><br>78% peds (27 pts)	68% (49/72) in total<br><br>63% homogenous<br>33% speckled<br>4% nucleolar<br><br>27/40 (68%) in childhood onset<br><br>22/32 (69%) in adult onset	11/12 (92%) ECDS pts had pos. ANA<br><br>ANA showed no other correlations (but AHA and anti-ssDNA did)
ANA	(30)<br><br>United States<br><br>2003-2013<br><br>**Pittsburgh** NRCOS	Indirect immunofluo-rescence (Hep2 cells) Assay standardized by duplication, High-PMT-Standard Dilution Series, natural cubic	**69 pts:**<br><br>-27 (39%) linear trunk<br>-14 (20%) linear head<br>-10 (14%) mixed<br>-8 (11%) generalized<br>-5 (7%) plaque<br>-4 (5%) deep<br>-1 (1%) Pansclerotic	Peds	69 peds and 71 healthy peds	9 (IQR: 7-11)	67%	22/62 (36%)	ANA pos. pts had significantly lower IL-17a, and IL-8

		splines and inter-panel and intra-assay control plasma samples.							
ANA	(28)<br><br>United States<br><br>2003-2013<br><br>**Pittsburgh** NRCOS	Indirect immunofluo-rescence (Hep2 cells)<br><br>Titer > 1:80<br><br>Assay standardized by fitting in logistic regression	**77 pts:**<br><br>-42 (54%) linear trunk<br>-12 (15%) linear face<br>-21 (27%) circumscribed superficial<br>-8 (10%) circumscribed deep<br>-10 (12%) generalized morphea<br>-12 (15%) mixed morphea<br>-2 (2%) pansclerotic<br>-1 (1%) eosinophilic fascitis	Peds	77 peds<br><br>35 re-lapse;<br>42 no relapse	10 (3.9) for pts with relapse<br>6.7 (3.7) for pts without relapse	70%	26/63 (41%)<br><br>Relapse 60% pos. ANA<br>Non-relapse 24% pos. ANA	Pts who relapsed were more likely to be ANA pos.<br><br>OR 4.8, CI [1.37-17.2]
ANA	(29)<br><br>United States<br><br>**Pittsburgh** NRCOS	Indirect immunofluo-rescence (Hep2 cells)<br><br>Titer > 1:80	**69 pts:**<br><br>-25 (37%) linear trunk/extremity<br>-15 (22%) Linear head<br>-16 (24%) generalized<br>-12 (17%) circumscribed superficial<br><br>46 healthy peds (case control)	Peds	69 peds<br><br>46 healthy peds	13.8 (+/- 4.7 SD)<br><br>Healthy: 12.1 (+/- 2.9 SD)	64% in LS<br><br>77% in healthy cont-rols	50% of total (35/69)<br><br>50% speckled<br>17% cytoplasmic<br>12% homogenous<br>10% nucleolar<br>9% centrosome<br><br>Titer 1:160 in 30%<br>Some had as high as 1:1520 (11%)	ANA titer correlated positively with disease severity, including number of site and LoSDI (skin damage score)
**European**									
ANA	(Suppl.ref.1)<br><br>Italy	Indirect immunofluo-rescence (Hep2 cells)<br>Assay standardized by double-blind study	**70 pts:**<br><br>-44 (63%) linear<br>-18 (26%) generalized<br>-8 (11%) mixed	Peds	70 peds:<br>46 MTX treated;<br>24 pla-cebo	N.D. (Range: 6-17)	71%	(43%)<br>30/70	4 pts in MTX treatment group had negative ANA at baseline but positive titers at the end;<br>1 MTX treated pt had positive ANA titer at baseline and a negative titer at the end
ANA	(9)<br><br>Germany	Indirect immunofluo-rescence (Hep2 cells)<br><br>ANA > 1:320 considered positive	**472 pts:**<br><br>**250 (53%) adult morphea:**<br>-60% plaque morphea<br>-27% generalized morphea	Adults and peds onset	381 adults<br><br>91 peds	46 average age total (4-88)	77% adults<br><br>88% peds	28/472 (5.9%)<br><br>ANA > 1:320 considered positive	ANA frequency was smaller compared to previous studies which may be attributed by different cut off levels for ANA pos.

			**91 peds:**<br>-33% linear extremities<br>-34% linear head						
ANA	(Suppl.ref.2)<br><br>Germany	ANA≥1:160 considered positive	**96 pts:**<br><br>-96 (100%) head and face LS	Adults and peds	60 adults<br><br>36 ped	21.9 (1-68)	71%	26/96 (27.1%) pts ANA pos.	ANA pos. seems to be higher in juvenile LS

Abbreviations-ANA= Antinuclear antibody, AHA= Anti histone antibody, ss DNA= single stranded DNA, IF= Immunofluorescence, LS= Localized Scleroderma, ECM= Extracutaneous manifestations, MAC= Morphea in Adults and Children, ECDS= en coup de sabre, Lin Extr= Linear Extremities, IQR= Interquartile Range, LoSDI= localized scleroderma skin damage index, mRSS= modified Rodnan skin score, M=Mixed, yr= year, CI= Confidence Interval, SD= Standard deviation, MTX= Methotrexate, ND= No Data, pts= patients, peds= pediatric, OR= Odds Ratio, pos=positive/positivity, PRES=Pediatric Rheumatology European Society, PRINTO=Pediatric Rheumatology International Trial Organization

Supplementary Table 2. Current antibody tests in morphea and clinical associations of their results: Anti-histone and anti-DNA antibodies (AHA, anti-ssDNA, anti-dsDNA).

Target	Ref Country and Cohort	Method details	# pts in cohort<br><br>subtype	Type of cohort	# pts, type, cont-rols	Mean age (range) in years	% Fe-male	% positive (total)	Clinical association
AHA	(37)	ELISA	**49 pts:**<br><br>15 generalized<br>22 LS<br>12 Morphea	Not specifi ed	22 healthy<br>12 DLE,<br>45 SSc	24.4yrs	N.D.	23/49 (47%) localized cohort<br><br>13/15 (87%) generalized	Presence of AHA strongly correlated with number of morphea lesions, number of lesions and number of involved areas of the body
AHA	(19)<br><br>United States<br><br>**MAC cohort**	ELISA kits(Orgentec Diagnostika)	**251 pts:**<br><br>251 screened<br><br>187 (74%) included in analysis	Adults and peds onset	110 adult<br><br>77 peds<br><br>>1800 healthy adults+ peds	10.1 (3.9) for peds onset<br>45.3 (15.6) for adult onset		12% (22/187)	Present with greater frequency in morphea than in nested case controls (p <0.001)<br><br>AHA was more frequently present in linear morphea pts (15/85) than in nonlinear subtypes (generalized 5/73, plaque 1/18) (p = 0.04)
AHA	(38)<br><br>United States<br><br>**Pittsburgh** 1991-2005	ELISA	**72 pts:**<br><br>-72 (100%) LS<br>-12 (16%) ECDS	Adults and peds onset	32 adults onset<br><br>40 peds onset	32.9 (14.5) adults onset<br><br>8.8 (3.9) peds onset	81%	27/70 (39%) in total<br><br>8/31 (26%) adults onset<br><br>19/39 (49%) peds onset	Mean AHA levels were higher in pts with more skin areas involved, active disease, and joint contractures; AHA pos. was also associated with involvement of more than 2 cutaneous sites
AHA	(30)<br><br>United States<br><br>**Pittsburgh** NRCOS	ELISA Assay standardized by duplication, High-PMT-Standard Dilution Series, natural cubic splines and inter-panel and intra-assay control plasma samples	**69 pts:**<br><br>-27 (39%) linear trunk<br>-14 (20%) linear head<br>-10 (14%) mixed<br>-8 (11%) generalized<br>-5 (7%) plaque<br>-4 (5%) deep<br>-1 (1%) pansclerotic	Peds	69 peds<br>71 healthy peds	9 (IQR: 7-11)	67%	19/59 (32%)	AHA pos. pts exhibited significantly elevated levels of IL-1 α, IL -1B, IL-2, IL-4, IL-10, IL-12p70, IL-13, IL17a, IL-17e, IL-17f, IL-22, IL-23, IL-31, IL-33, IFN- γ, IFN- α2, GM-CSF, VEGF<br><br>Generally correlated with clinical

									characteristics of disease severity e.g. total number of lesions and total body areas affected
AHA	(28)<br><br>United States<br><br>**Pittsburgh** NRCOS	ELISA Assay standardized by fitting in logistic regression	**77 pts:**<br><br>See above subtype	Peds	77 peds 35 Relaps e 42 No relapse	10 (3.9) for pts with relapse 6.7 (3.7) for pts without relapse	70%	26/70 (37%)	AHA pos. did not predict relapse
AHA	(9)<br><br>Germany	N.D.	**472 pts:**<br><br>**250 (53%) adult morphea:**<br>-60% plaque morphea<br>-27% generalized morphea<br><br>**91 peds:**<br>-33% linear extremities<br>-34% linear head	Adults and peds onset	381 adults<br><br>91 peds	46 (4-88)	77% adult<br><br>88% peds	5/472 (1%)	Not discussed
Anti ssDNA	(19)<br><br>United States<br><br>**MAC cohort**	ELISA kits(Orgentec Diagnostika	**251 pts:**<br><br>251 (100%) screened<br><br>187 (74%) included in analysis	Adults and peds onset	110 adults<br><br>77 peds<br><br>>1800 healthy adults+ peds	45.3 (15.6) adults onset<br><br>10.1 (3.9) peds onset		15/187 (8%)	Not discussed
Anti-ssDNA	(40)<br><br>United States<br><br>**MAC cohort**	N.D.	**369**(HLA I+HLAII) **pts:**<br><br>-95 (39%) linear<br>-75 (31%) generalized<br>-245 (100%) plaque<br>-21 (8%) other	Adults and peds onset	1677 (HLAI + HLAII)	27 (12-37)<br><br>Not distinguis hed between peds and adult	82.5%	13/211(6%)	Not analyzed due to infrequency
Anti-ssDNA	(27)<br><br>United States<br><br>**Pittsburgh** 1962-1983	N.D.	**53 pts**	Adults and peds onset	44 adults onset<br><br>9 peds onset	44 pts <25 yr at age of onset<br><br>9 pts >25 yr at age of onset	80%	20/39 (51%)	More common in pts with joint contractures and disease duration > 2 years.<br><br>Pts with higher level of a-ssDNA had more extensive disease
Anti-ssDNA	(38)<br><br>United States<br><br>**Pittsburgh** 1991-2005	ELISA	**72 pts:**<br><br>-72 (100%) LS<br><br>-12 (16%) ECDS	Adults and peds onset	32 adults onset<br><br>40 peds onset	32.9 (14.5) adults onset<br><br>8.8 (3.9) peds onset	81%	20/70 (29%)<br><br>6/31 (19%) adults onset<br><br>14/39 (36%) peds onset	Higher mean anti-ssDNA levels were detected in LiSCl pts with more extensive skin involvement

									and active disease; also associated with involvement of more than 2 cutaneous sites.
Anti-ssDNA	(30)<br><br>United States<br><br>**Pittsburgh NRCOS**	ELISA >19 U/mL considered positive; Assay standardized by duplication, High-PMT-Standard Dilution Series, natural cubic splines and inter-panel and intra-assay control plasma samples	**69 pts:**<br><br>See above subtypes	Peds	69 peds<br><br>71 healthy pedss	9 (IQR: 7-11)	67%	17/57 (30%)	Elevated anti-ssDNA associated with IFN- α2 and IL-33.
Anti-ssDNA	(28)<br><br>United States<br><br>**Pittsburgh NRCOS**	ELISA Assay standardized by fitting in logistic regression	**77 pts:**<br><br>See above subtypes	Peds	77 peds<br>35 Relap-se<br>42 No relapse	10 (3.9) for pts with relapse<br>6.7 (3.7) for pts without relapse	70%	31/71 (44%)	ssDNA pos. did not predict relapse
Anti-ssDNA and AHA	(38)<br><br>United States<br><br>**Pittsburgh**	ELISA	**72 pts:**<br><br>-72 (100%) LS<br><br>-12 (16%) ECDS	Adults and peds onset	32 adults onset<br><br>40 peds onset	32.9 (14.5) adults onset<br><br>8.8 (3.9) peds onset	81%	16/70 (23%)<br><br>4/31 (13%) adults onset<br><br>12/39 (31%) peds onset	Presence of anti-ssDNA and AHA was associated with joint contractures in childhood onset pts
Anti-dsDNA	(13, 18)<br><br>**Cohorts:**<br>38 European;<br>12 North American;<br>11 South American;<br>8 Asian;<br>1 Australian	Retrospective collection of patients' data from PRES and PRINTO	**750 pts:**<br><br>-489 (65%) linear<br>-113 (15%) linear head<br>-194 (26%) plaque<br>-51 (7%) generalized<br>-16 (2%) deep<br><br>-514 (68%) skin involvement only<br>-157 (21%) with Extracutaneous manifestations (ECM)	Peds	750 peds	7.3 (0-16)	70.5%	16/382 (4.2%) overall<br><br>-subtypes:<br>12/246 (4.9%) in linear<br>3/105 (2.9%) in plaque<br>0/26 (0%) in generalized<br>1/5 (20%) in deep<br><br>-ECM:<br>13/320 (4%) in pts with skin involvement only<br>3/62 (4.8%) in pts with	Largest LS cohort to date with dsDNA evaluated in 382 subjects<br><br>dsDNA pos. not significantly different in subtypes<br><br>dsDNA was not significantly different in pts with ECM than those with skin involvement only<br><br>None of pts with ds-DNA had SLE features and none had a

								EC involvement	family history of SLE
Anti-dsDNA	(Suppl.ref.3)<br><br>Canada	N.D.	**52 pts:**<br><br>-65% with affected limbs<br>-35% with affected face	Peds	52 peds	7.3 (8.5 mos to 16.2 yrs)	69%	8/52 (15%)	Not discussed
Anti-dsDNA	(10)<br><br>United States	N.D.	**245 pts:**<br><br>See above subtypes	Adults and peds	123 adults<br><br>122 peds	44.7 adult<br><br>8.7 peds	84% adults<br><br>79% peds	3/19 (15.8%) overall<br><br>1/3 (33.3%) in linear<br>1/6 (16.7%) in generalized<br>1/3 (33.3%) in mixed	None of pts developed SLE after 1 year follow-up
Anti-dsDNA	(Suppl.ref.1)<br><br><br>Italy	Assay standardized by double-blind study	**70 pts:**<br><br>-44 (63%) linear<br>-18 (26%) generalized<br>-8 (11%) mixed	Peds	70 peds<br>46 Methotrexate treated<br>24 Placebo	N.D. (6-17)	71%	1/70 (1%)	1 pt with positive anti-dsDNA tested negative at the end of the trial
Anti-dsDNA	(9)<br><br>Germany	N.D.	**472 pts:**<br><br>**250 (53%) adult morphea:**<br>-60% plaque morphea<br>-27% generalized morphea<br><br>**91 peds:**<br>-33% linear extremities<br>-34% linear head	Adults and peds onset	381 adults<br><br>91 peds	46 (4-88)	77% adults<br><br>88% peds	11/472 (2.3%)	Not discussed

Abbreviations-AHA= Anti histone antibody, Anti-ss DNA=Anti single stranded DNA, ECDS= en coup de sabre, Anti-ds DNA=Anti double stranded DNA, SLE= Systemic lupus erythematosus, ELISA = Enzyme-linked immunosorbent assay, LS= Localized Scleroderma, ECM= Extracutaneous manifestations, MAC= Morphea in Adults and Children, GM-CSF= Granulocyte-macrophage colony-stimulating factor, VEGF= Vascular endothelial growth factor, NRCOS= National Registry of Childhood Onset Scleroderma, LiSCL= linear scleroderma Database IQR= Interquartile Range, yr= year, ND= No Data, pts= patients, peds= pediatric; pos=positive/positivity, DLE=Discoid Lupus Erythematosus, SSc=Systemic Sclerosis.

Supplementary Table 3. Current antibody tests in morphea and clinical associations of their results: other antibodies.

**Target**	**Ref Country and Cohort**	**Method details**	**# pts in cohort**<br><br>**subtype**	**Type of cohort**	**# pts, type, controls**	**Mean age (range) in years**	**% Female**	**% positive (total)**	**Clinical association**
ACA	(13, 18)<br><br>**Cohorts:**<br>38 European;<br>12 North American;<br>11 South American;<br>8 Asian;<br>1 Australian	Retrospective collection of patients' data from PRES and PRINTO	**750 pts:**<br><br>-489 (65%) linear<br>-113 (15%) linear head<br>-194 (26%) plaque<br>-51 (7%) generalized<br>-16 (2%) deep<br><br>-514 (68%) skin involvement only<br>-157 (21%) with Extracutaneous manifestations (ECM)	Peds	750 peds	7.3 (0-16)	70.5%	4/240 (1.7%) overall<br><br>-subtypes:<br>3/162 (1.9%) in linear<br>0/45 (0%) in plaque<br>1/25 (4%) in generalized<br>0/8 (0%) in deep<br><br>-ECM:<br>2/169 (1.2%) in pts with skin involvement only<br>2/71 (2.8%) in pts with EC involvement	Largest LS cohort to date with dsDNA evaluated in 240 subjects<br><br>ACA pos. not significantly different in subtypes<br><br>ACA was not significantly different in pts with ECM vs. those with skin involvement only<br><br>None of pts with ACA pos. had SSc features and none had a family history of SSc
ACA	(10)<br><br>United States<br><br>**MAC cohort**	N.D.	**245 pts:**<br><br>See above subtypes	Adults and peds	123 adults<br><br>122 peds	44.7 adults<br><br>8.7 peds	84% adults<br><br>79% peds	1/11 (9.1%) overall<br><br>1/2 (50%) generalized	Not discussed
ACA	(29)<br><br>United States<br><br>**NRCOS cohort**	ALBIA MagPix® (Luminex™)	**69 pts:**<br><br>See subtypes above	Peds	69 peds<br><br>46 healthy peds	N.D.	N.D.	6/69 (9%)	No associations
CENP A	(29)<br><br>United States	LIA(Euroimmun, Germany)	**69 pts:**<br><br>See subtypes above	Peds	69 peds<br><br>46 healthy peds	N.D.	N.D.	10/69 (14%)	Joint contracture, pain at lesion, subcutaneous atrophy, MSK involvement
CENP B	(29)<br><br>United States	LIA(Euroimmun, Germany)	**69 pts:**<br><br>See subtypes above	Peds	69 peds<br><br>46 healthy peds	N.D.	N.D.	4/69 (6%)	Lesion pain, tingling
ACA	(9)<br><br>Germany	N.D.	**472 pts:**<br><br>See subtypes above	Adults and peds onset	381 adults<br><br>91 peds	46 (4-88)	77% adults<br><br>88% peds	3/472 (0.6%)	Not discussed

Anti-Scl70	(13, 18)<br><br>**Cohorts:**<br>38 European;<br>12 North American;<br>11 South American;<br>8 Asian;<br>1 Australian	Retrospective collection of patients' data from PRES and PRINTO	**750 pts:**<br><br>-489 (65%) linear<br>-113 (15%) linear head<br>-194 (26%) plaque<br>-51 (7%) generalized<br>-16 (2%) deep<br><br>-514 (68%) skin involvement only<br>-157 (21%) with Extracutaneous manifestations (ECM)	Peds	750 peds	7.3 (0-16)	70.5%	12/378 (3.2%) overall<br><br>-subtypes:<br>7/250 (2.8%) in linear<br>4/88 (4.5%) in plaque<br>0/29 (0%) in generalized<br>1/11 (9%) in deep<br><br>-ECM:<br>9/303 (3.0%) in pts with skin involvement only<br>3/75 (4%) in pts with EC involvement	Largest LS cohort to date with SCl-70 eval in 378 subjects<br><br>Scl-70 not tested for significantly different in subtypes<br><br>SCl-70 was not significantly different in pts with ECM than those with skin involvement only<br><br>None of pts with SCl-70 had SSc features and none had a family history of SSc
Anti-Scl70	(Suppl.ref.3)<br><br>Canada	N.D.	**52 pts:**<br><br>-65% with affected limbs<br>-35% with affected face	Peds	52 peds	7.3 (8.5 mos to 16.2 yrs)	69%	1/52 (2%)	Not discussed
Anti-Scl70	(10)<br><br>United States	N.D.	**245 pts:**<br><br>See above subtypes	Adults and peds	123 adults<br><br>122 peds	8.7 peds 44.7 adult	84% adults 79% peds	1/39 (2.6%) overall<br><br>1/8 (12.5%) in generalized	Not discussed
Anti-Scl70	(29)<br><br>United States<br><br>**Pittsburgh** NRCOS	ALBIA MagPix® (Luminex™) and LIA (Euroimmun, Germany)	**69 pts:**<br><br>See subtypes above	Peds	69 peds	N.D.	N.D.	6/69 (9%) via ALBIA<br>7/69 (10%) via LIA EUROIMMUN	Anti-Scl pos. associated with skin texture, pain at lesion, subcutaneous atrophy, joint contracture
Anti-Scl70	(9)<br><br>Germany	N.D.	**472 pts:**<br><br>See subtypes above	Adults and peds onset	381 adults<br><br>91 peds	46 (4-88)	77% adults<br><br>88% peds	4/472 (0.8%)	Not discussed
Anti snRNP	(10)<br><br>United States	N.D.	**245 pts:**<br><br>See Subtypes above	Adults and peds	123 adults 122 peds	44.7 adults<br><br>8.7 peds	84% adults<br><br>79% peds	1/21 (4.8%)<br><br>1/5 (20%) in generalized	Not discussed
SM-RNP	(29)<br><br>United States	ADIS	**69 pts:**<br><br>See subtypes above	Peds	69 peds	N.D.	N.D.	11/69 (16%)	Shiny lesion, hyperpigmented, renal involvement

Anti-Sm	(Suppl.ref.3)<br><br>Canada	N.D.	**52 pts:**<br><br>-34 (65%) with affected limbs<br>-18 (35%) with affected face	Peds	52 peds	7.3 (8.5 mos to 16.2 yrs)	69%	1/52 (2%)	Not discussed
Anti-Sm	(29)<br><br>United States	ADIS	**69 pts**	Peds	69 peds	N.D.	N.D.	8/69 (12%)	Shiny lesion, hypo- and hyperpigmented
Anti-Sm	(9)<br><br>Germany	N.D.	**472 pts:**<br><br>See subtypes above	Adults and peds onset	381 adults<br><br>91 peds	46 (4-88)	77% adults<br><br>88% peds	1/472 (0.2%)	Not discussed
Anti-Sm	(9)<br><br>Germany	N.D.	**96 pts:**<br><br>96 (100%) with head and face LS	Adults and peds	N.D.	21.9 (1-68)	71%	11/96 (11.5%)	Not discussed
PM-75	(29)<br><br>United States	LIA (Euroimmun, Germany)	**69 pts:**<br><br>See subtypes above	Peds	69 peds	N.D.	N.D.	4/69 (6%)	Shiny lesion, hypopigmented, pulmonary involvement
PM-SCL	(29)<br><br>United States	ALBIA MagPix® (Luminex™) and LIA (Euroimmun, Germany)	**69 pts:**<br><br>See subtypes above	Peds	69 peds	N.D.	N.D.	7/69 (10%) via ALBIA 10/69 (14%) via LIAEUROIMMUN	Subcutaneous trophy, pain at onset, endocrine involvement
Anti Sjogren syndrome associated antigen B antiboides and RF	(Suppl.ref.3)<br><br>Canada	N.D.	**52 pts:**<br><br>65% with affected limbs<br>35% with affected face	Peds	52 peds	7.3 (8.5 mos to 16.2 yrs)	69%	1/52 (2%)	Not discussed
RO-52	(29)<br><br>United States	LIA(Euroimmun, Germany) and ADIS	**69 pts:**<br><br>See subtypes above	Peds	69 peds	N.D.	N.D.	7/69 (10%) via LIA EUROIMMUN<br>21/69 (30%) via ADIS ENA Profile	Thick skin, joint contracture, neurologic involvement
SSA-60	(29)<br><br>United States	ADIS	**69 pts:**<br><br>See subtypes above	Peds	69 peds	N.D.	N.D.	10/69 (14%)	Skin thickness
SSB	(29)<br><br>United States	ADIS	**69 pts:**<br><br>See subtypes above	Peds	69 peds	N.D.	N.D.	5/69 (7%)	Tight skin, hypopigmented
RF	(13, 18)<br><br>**Cohorts:**<br>38 European;<br>12 North American;<br>11 South American;<br>8 Asian;<br>1 Australian	Retrospective collection of patients' data from PRES and PRINTO	**750 pts:**<br><br>-489 (65%) linear<br>-113 (15%) linear head<br>-194 (26%) plaque<br>-51 (7%) generalized<br>-16 (2%) deep<br><br>-514 (68%) skin involvement only	Peds	750 peds	7.3 (0-16)	70.5%	74/464 (15.9%) overall<br><br>-subtypes:<br>59/311 (19%) in linear<br>12/117 (10.3%) in plaque<br>4/30 (13.3%) in generalized	Largest LS cohort to date with RF eval in 464 subjects<br><br>RF not tested for significantly different in subtypes<br><br>RF pos. was higher in pts with EC

			-157 (21%) with Extracutaneous manifestations (ECM)					16 (17%) in deep<br><br>-ECM:<br><br>46/349 (13.2%) in pts with skin involvement only<br>28/115 (24.3%) in pts with EC involvement	involvement than those with only skin involvement<br><br>RF was positive in higher percentage of pts with arthritis compared to those without arthritis.<br><br>Suggests that RF is a major clinical marker
RF	(9)<br><br>Germany	N.D.	**472 pts:**<br><br>See subtypes above	Adults and peds onset	381 adults<br><br>91 peds	46 (4-88)	77% adults<br><br>88% peds	18/472 (3.8%)	Not discussed
RF	(Suppl.ref.1)<br><br>Italy	Assay standardized by double-blind study	**70 pts:**<br><br>-44 (63%) linear<br>-18 (26%) generalized<br>-8 (11%) mixed	Peds	70 peds<br>46 Methotrexate treated<br>24 Placebo	N.D. (6-17)	71%	2/70 (3%)	2 pts with positive RF tested negative at the end of the trial
RF	(Suppl.ref.4)<br><br>Italy	N.D.	**58 pts:**<br><br>-37 (64%) linear<br>-13 (22%) generalized<br>-8 (14%) mixed	Peds	58 peds<br>48 MTX responders<br>10 MTX refractory	9.4 (N.D.)	72	2/58 (3.4%)<br><br>2/48 (4.2%) in Methotrexate Responders	Not discussed

Abbreviations- ACA= Anti-centromere antibody, ECM= Extracutaneous manifestations, ALBIA= Addressable Laser Bead Immunoassay, Ssc= Systemic Sclerosis, NRCOS= National Registry of Childhood Onset Scleroderma, CENP= centromere, LIAEUROIMMUN= line immunoassay (LIA) (Euroimmun, Germany), MSK= Medullary Sponge Kidney, LS= Localized Scleroderma, Anti-Scl-70= anti-topoisomerase I, Anti-snRNP= Anti- small nuclear ribonucleoproteins, SM-RNP= Smith ribonucleoproteins, PM-75= polymyositis-75, PM-Scl= polymyositis-scleroderma, RF= Rheumatoid factor, SSA-60= Sjogren's-syndrome-related antigen-60, mos=months, yr= year, ND= No Data, pts= patients, peds= pediatric, pos=positive/positivity, ADIS=Alpha Diagnostic International ADI San Antonio Texas, MTX= Methotrexate, PRES=Pediatric Rheumatology European Society, PRINTO=Pediatric Rheumatology International Trial Organization.

Bibliography

For references #1-70, see main paper;

Supplementary ref.1: Zulian F, Martini G, Vallongo C, Vittadello F, Falcini F, Patrizi A, Alessio M, La Torre F, Podda RA, Gerloni V, et al. Methotrexate treatment in juvenile localized scleroderma: a randomized, double-blind, placebo-controlled trial. *Arthritis Rheum* (2011) 63:1998–2006. doi:10.1002/art.30264

Supplementary ref.2: Kreuter A, Mitrakos G, Hofmann SC, Lehmann P, Sticherling M, Krieg T, Lahner N, Tigges C, Hunzelmann N, Moinzadeh P. Localized Scleroderma of the Head and Face Area: A Retrospective Cross-sectional Study of 96 Patients from 5 German Tertiary Referral Centres. *Acta Derm Venereol* (2018) 98:603–605. doi:10.2340/00015555-2920

Supplementary ref.3: Piram M, McCuaig CC, Saint-Cyr C, Marcoux D, Hatami A, Haddad E, Powell J. Short- and long-term outcome of linear morphoea in children. *Br J Dermatol* (2013) 169:1265–1271. doi:10.1111/bjd.12606

Supplementary ref.4: Zulian F, Vallongo C, Patrizi A, Belloni-Fortina A, Cutrone M, Alessio M, Martino S, Gerloni V, Vittadello F, Martini G. A long-term follow-up study of methotrexate in juvenile localized scleroderma (morphea). *J Am Acad Dermatol* (2012) 67:1151–1156. doi:10.1016/j.jaad.2012.03.036
